# Identifying Distinct Molecular Subtypes and Establishing a Prognostic Framework for DLBCL Patients via Multiomics Analysis and Machine Learning Approaches

**DOI:** 10.1155/humu/7614954

**Published:** 2026-07-06

**Authors:** Hongyu Shen, Jinbo Lu, Qi Yan, Jinjiang Chou, Xiao Liang, Weifei Fan, Lei Fan

**Affiliations:** ^1^ Department of Hematology, The First Affiliated Hospital With Nanjing Medical University, Jiangsu Province Hospital, Nanjing, Jiangsu, China, jsph.net; ^2^ Jiangsu Province Engineering Research Center of Cell and Gene Therapy for hematologic and lymphoid Diseases, Nanjing, Jiangsu, China; ^3^ Department of Hematology, Yancheng No. 1 People′s Hospital, Yancheng, Jiangsu, China; ^4^ Department of Oncology, Huai′an Hospital of Hua′an City, Huai′an Cancer Hospital, Huai′an, Jiangsu, China; ^5^ Department of Cell and Molecular Biology, Karolinska Institutet, Stockholm, Sweden, ki.se; ^6^ Department of Oncology, The Affiliated Jiangyin Hospital of Nantong University, Jiangyin, Jiangsu, China; ^7^ Department of Hematology and Oncology, Jiangsu Province Geriatric Hospital, Nanjing, Jiangsu, China

**Keywords:** diffuse large B-cell lymphoma (DLBCL), machine learning approaches, molecular subtyping, prognostic framework, single-cell RNA sequencing (scRNA-seq)

## Abstract

Diffuse large B‐cell lymphoma (DLBCL) is characterized by profound heterogeneity that underpins varied clinical outcomes. To decipher this complexity, we performed an integrated single‐cell and genomic analysis. Using scRNA‐seq data (GSE182434), we identified six distinct malignant B‐cell subclusters (MB1‐MB6) within the DLBCL ecosystem. Cell–cell communication analysis revealed intricate interaction networks, particularly involving the MIF and Complement pathways. Prognostic analysis of bulk transcriptomic data (GSE32918) identified the MB5‐related gene signature as the most critical factor associated with poor overall survival. This MB5 subgroup was associated with enhanced proliferative processes, a higher tumor mutational burden, and specific comutations. Leveraging MB5 marker genes, we developed and validated a robust CoxBoost‐RSF machine‐learning model that effectively stratified patient risk in independent cohorts. Our study defines the MB5 malignant B‐cell subgroup as a key driver of DLBCL aggressiveness and provides both a novel prognostic biomarker and a framework for personalized therapeutic targeting.

## 1. Introduction

Diffuse large B‐cell lymphoma (DLBCL) is the most common subtype of non‐Hodgkin lymphoma, characterized by its clinical and molecular heterogeneity, which contributes to varied treatment responses and outcomes [1, 2]. This heterogeneity is evident in the diverse range of symptoms, disease progression rates, and treatment responses observed among patients with DLBCL. Despite recent therapeutic advancements, including the incorporation of targeted therapies and immunoconjugates, a significant portion of patients still face relapse or resistance to treatment [3, 4]. This clinical challenge underscores the need for a deeper understanding of the disease′s complexity and the development of more effective, personalized treatment strategies [5]. The molecular heterogeneity of DLBCL is reflected in the diverse genetic alterations observed across tumor samples. These alterations can include mutations, alterations in gene expression, and chromosomal rearrangements that affect key signaling pathways involved in cell growth, survival, and immune evasion [6–8]. The presence of these diverse molecular profiles can lead to different disease subtypes, each with its own distinct prognosis and response to treatment. Therefore, understanding the molecular underpinnings of DLBCL is crucial for developing novel therapeutic approaches that target the specific genetic and cellular features of each patient′s tumor.

The advent of single‐cell RNA sequencing (scRNA‐seq) has revolutionized our ability to dissect the cellular heterogeneity within tumors [9–11]. This technology allows for the characterization of individual cells, revealing distinct subpopulations of malignant B cells and their interactions with the tumor microenvironment. By identifying these subgroups, we can potentially develop novel prognostic markers and therapeutic targets. In this study, we leveraged scRNA‐seq data from the GEO database to explore the cellular heterogeneity in DLBCL. We focused on dataset GSE182434 to identify distinct subgroups of malignant B cells and dataset GSE32918 to construct prognostic models. Additionally, we integrated somatic mutation data and gene expression data from The Cancer Genome Atlas (TCGA) to explore the genetic landscape of DLBCL.

Our analysis involved rigorous quality control, data normalization, and clustering to stratify cells into principal cell types. We identified six distinct malignant B‐cell subclusters (MB1–MB6) based on their expression profiles. Pseudotime analysis was used to reconstruct cell differentiation trajectories and understand the dynamic relationships among these subgroups.

We employed the CellChat algorithm to map the communication network among different cell subpopulations, providing insights into the cellular crosstalk within the tumor microenvironment. Functional enrichment analysis using the Metascape platform further characterized the biological functions of each subgroup.

The prognostic significance of these subgroups was evaluated using Gene Set Variation Analysis (GSVA), revealing that the MB5 subgroup had the highest predictive value for patient survival. We also conducted Weighted Gene Coexpression Network Analysis (WGCNA) to explore the gene expression characteristics of the MB5 subgroup, identifying core modules and hub genes.

Analysis of the tumor mutational burden (TMB) and proliferation capacity in the TCGA‐DLBCL cohort showed that patients with high MB5 signature scores had significantly higher TMB and proliferation scores, suggesting a link between high MB5 subgroup scores and enhanced tumor cell proliferation capacity.

Finally, we constructed prognostic models using machine learning ensembles based on MB5 marker genes, with the CoxBoost‐RSF ensemble demonstrating the highest predictive performance for overall survival in DLBCL patients.

This study provides a comprehensive analysis of the cellular and molecular heterogeneity within DLBCL, identifying the MB5 subgroup as a critical factor affecting patient prognosis. Our findings highlight the MB5 subgroup as a potential therapeutic target and pave the way for future studies to improve patient outcomes in DLBCL.

## 2. Materials and Methods

### 2.1. Data Sources

This study utilized scRNA‐seq data of DLBCL from the Gene Expression Omnibus (GEO) database, specifically dataset GSE182434 [12], as the discovery cohort for cell heterogeneity analysis. Additionally, the gene expression profiling dataset GSE32918 was downloaded to construct prognostic models [13, 14], and somatic mutation data, along with gene expression data for DLBCL (TCGA‐DLBCL), were obtained from TCGA database [15]. Four DLBCL cohorts of whole‐exome sequencing (WES) data were obtained from the cBioPortal database for analyses of mutational frequency and hotspots [16–19]. We retrieved the precomputed immune signature scores and immune cell fraction estimates from a pan‐cancer study by Thorsson et al. [20].

### 2.2. scRNA‐seq Data Analysis

#### 2.2.1. Data Preprocessing and Quality Control

The raw gene expression matrix was imported, and Seurat objects were constructed using R software (v4.2.0) and the Seurat package (v4.3.0) [21, 22]. Cells with fewer than 200 or more than 2500 genes or with mitochondrial gene content exceeding 10% were rigorously filtered out to ensure high‐quality data. Ultimately, 14,322 high‐quality cells were obtained for subsequent analysis.

#### 2.2.2. Data Normalization, Dimensionality Reduction, and Clustering

Gene expression data were normalized using the “LogNormalize” method, and highly variable genes were selected for principal component analysis (PCA). The “JackStraw” method was employed to identify significant principal components, followed by UMAP nonlinear dimensionality reduction. Unsupervised clustering was performed in the UMAP space based on graph clustering algorithms, and cell types were annotated using known cell marker genes.

#### 2.2.3. Identification and Subgroup Analysis of Malignant B Cells

Malignant B cells were identified from the total cell population based on the expression of B cell marker genes CD19 and MS4A1 [12]. After extracting this cell subset, the normalization, dimensionality reduction, and clustering processes were repeated to identify subgroups of internal heterogeneity. The “FindAllMarkers” function (Wilcoxon rank‐sum test) was used to identify marker genes for each subgroup.

#### 2.2.4. Pseudotime Analysis

Pseudotime analysis of malignant B cells was conducted using the Monocle2 R package to reconstruct cell differentiation trajectories and clarify the dynamic relationships among subgroups during development [23].

#### 2.2.5. Cell–Cell Interaction Analysis

We conducted a cell–cell interaction analysis using the CellChat algorithm, a computational framework designed to dissect the communication network among different cell types. The algorithm was applied to processed scRNA‐seq data to identify and quantify interactions among cell populations. By mapping ligand‐receptor pairs and their inferred signaling activities, CellChat allowed us to construct a comprehensive interaction network [24].

### 2.3. Functional Enrichment Analysis

For genes specifically highly expressed in each malignant B cell subgroup, Gene Ontology (GO) biological process enrichment analysis was performed using the Metascape online platform [25]. The significance threshold was set at a minimum *p* value < 0.01, a cumulative hypergeometric test *p* value < 0.05, and a minimum enrichment factor > 1.5.

### 2.4. Construction and Validation of Prognostic Models

#### 2.4.1. GSVA Scoring

The Top 500 significant marker genes from each malignant B cell subgroup were integrated to construct characteristic gene sets for each subgroup. The GSVA algorithm [26] was used to analyze bulk RNA sequencing data from the GSE32918 dataset, calculating enrichment scores for each subgroup characteristic set in each sample.

#### 2.4.2. Survival Analysis

The importance of each subgroup characteristic score on patient overall survival was assessed using a random survival forest model. The optimal cutoff value for the MB5 subgroup characteristic score was determined using the maximally selected rank statistics, and patients were divided into high‐ and low‐scoring groups accordingly. Survival curves were plotted using the Kaplan–Meier method, and the log‐rank test was used to compare survival differences between the two groups. To assess the predictive performance of our prognostic models over time, we performed time‐dependent receiver operating characteristic (tROC) analysis using the survivalROC package in R. We calculated AUC values at 1, 2, and 3 years to evaluate the model′s effectiveness over different follow‐up periods.

#### 2.4.3. Machine Learning Approaches

For the construction of prognostic models, we used 101 machine learning ensemble methods on the MB5 marker genes. The GSE32918 dataset served as the training cohort, whereas the TCGA‐DLBCL dataset was used for testing. Each model′s performance was assessed using the concordance index (C‐index) [27]. Among these ensembles, the CoxBoost‐RSF (CoxBoost combined with random survival forest) model achieved the highest predictive performance. Specifically, CoxBoost was first applied to perform feature selection and estimate initial coefficients via penalized partial likelihood, followed by a random survival forest to refine risk scores and capture nonlinear effects. The final CoxBoost‐RSF model retained 13 MB5 marker genes and was validated in the independent TCGA‐DLBCL cohort.

### 2.5. Single‐Cell Weighted Gene Coexpression Network Analysis (hdWGCNA)

Gene expression data related to the MB5 subgroup were extracted from malignant B cells, and a coexpression network was constructed using the “hdWGCNA” R package [28]. After soft‐thresholding power filtering, a power value of 3 was determined to build a scale‐free network. Dynamic tree cutting was used to identify coexpression modules, and the correlation between module eigengenes and each malignant B cell subgroup was calculated. The core module most relevant to the MB5 subgroup was selected, and hub genes within the module were identified using gene connectivity (kME).

### 2.6. TMB and Proliferation Capacity Analysis

For the TCGA‐DLBCL cohort, mutation data were processed and analyzed using the Maftools R package to visualize and statistically evaluate the genetic alterations [29]. The somaticInteractions function in Maftools was used to identify potential interactions among somatic mutations, which could provide insights into the complex genetic interactions in DLBCL tumors. The lollipopPlot function was also used to graphically represent the distribution of mutations across the genome, highlighting mutational hotspots and mutation types. Furthermore, we calculated the nonsilent TMB for each patient, which quantifies the total number of nonsilent mutations in the tumor genome′s coding regions. Additionally, a tumor proliferation score was determined based on the expression levels of genes known to be associated with cell proliferation [20].

To statistically compare TMB and proliferation scores between patients with high and low MB5 signature scores, we used the Wilcoxon test. The mutation frequency of specific genes was compared using Fisher′s exact test.

### 2.7. Protein–Protein Interaction (PPI) Analysis

We used the String database to perform a PPI network analysis of the core genes in the MB5 subgroup. This online tool reveals protein interactions based on experimental data, databases, and coexpression evidence [30].

### 2.8. Drug Sensitivity Estimation

To identify potential drug options associated with the risk groups defined by the gene signature, we performed drug sensitivity analyses. The oncoPredict R package (v0.2) was used to estimate drug sensitivity for each sample, leveraging the precomputed pharmacogenomic data from the Genomics of Drug Sensitivity in Cancer (GDSC2) database. The package infers the half‐maximal inhibitory concentration (IC50) values from sample gene expression profiles. For each drug, estimated IC50 values were compared between the low‐risk and high‐risk groups. Drugs with significantly lower estimated IC50 (indicating higher predicted sensitivity) in the high‐risk group were prioritized.

### 2.9. Statistical Analysis

All statistical analyses were performed in the R language environment (Version 4.2.0). For survival analysis, differences between Kaplan–Meier survival curves were assessed using the log‐rank test. Comparisons of continuous variables between two groups were conducted using Student′s *t*‐test if the data met assumptions of normal distribution and homogeneity of variances. If these assumptions were violated, the nonparametric Mann–Whitney *U* test (Wilcoxon rank‐sum test) was applied. For comparisons involving categorical variables, such as mutation frequencies between groups, Fisher′s exact test or the chi‐squared test was used as appropriate. A two‐sided *p* value < 0.05 was considered statistically significant.

## 3. Results

### 3.1. Single‐Cell Sequencing Analysis Reveals Heterogeneous Subgroups of Malignant B Cells in DLBCL

**Figure 1:**
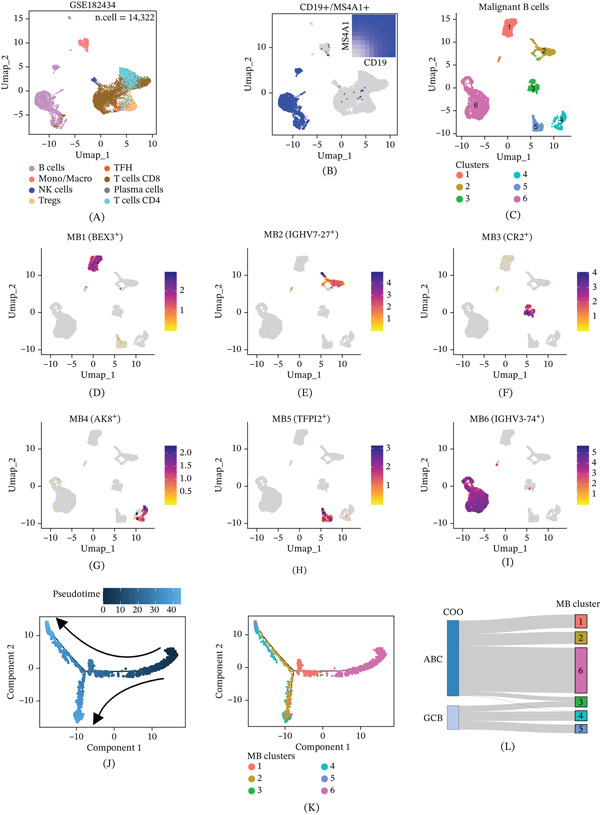
Single‐cell transcriptomic profiling identifies six malignant B‐cell subclusters in DLBCL. (A) UMAP visualization of 14,322 single cells from DLBCL samples, color‐coded by eight major cell types. (B) UMAP plot highlighting the malignant B‐cell population based on the expression of CD19 and MS4A1. (C) UMAP plot showing six distinct malignant B‐cell subclusters (MB1‐MB6) identified from reclustering. (D–I) UMAP plots depicting the expression of specific marker genes for each subcluster: BEX3 (MB1), IGHV7‐27 (MB2), CR2 (MB3), AK8 (MB4), TFPI2 (MB5), and IGHV3‐74 (MB6). (J) Pseudotime trajectory analysis of malignant B cells. (K) Positioning of the six MB subclusters along the pseudotime trajectory. (L) Sankey diagram illustrating the cellular origins (ABC/GCB subtypes) of the six malignant B‐cell subclusters.

This study obtained scRNA‐seq data of DLBCL from the GEO database (GSE182434). Following rigorous quality‐control measures and normalization processes, the analysis encompassed a total of 14,322 cells. UMAP was applied for dimensionality reduction, and these cells were stratified into eight principal cell types: B cells, monocytes/macrophages, natural killer (NK) cells, regulatory T cells (Tregs), follicular helper T cells (TFH), CD8+ T cells, plasma cells, and CD4+ T cells (Figure 1A). The malignant B‐cell subpopulation was accurately pinpointed within the UMAP plot based on the expression patterns of CD19 and MS4A1 (Figure 1B). Further analysis involved extracting 3436 malignant B cells from the total cell population to enable more granular subclustering, culminating in the identification of six distinct malignant B‐cell subclusters (MB1–MB6) (Figure 1C). Figure 1D–I illustrates the expression profiles of specific marker genes for each subcluster, as depicted by UMAP plots: MB1 (BEX3), MB2 (IGHV7‐27), MB3 (CR2), MB4 (AK8), MB5 (TFPI2), and MB6 (IGHV3‐74), which are crucial for understanding the biological characteristics and functions of each subcluster. Figure 1J presents a pseudotime analysis of malignant B cells, revealing dynamic processes of cellular differentiation and development. Figure 1K displays the positioning of the six malignant B‐cell subclusters within the pseudotime trajectory. Figure 1L shows a Sankey diagram that illustrates the relationships among the six malignant B‐cell subclusters and their cellular origins. Specifically, subclusters MB1, MB2, and MB6 predominantly arise from ABC, whereas subclusters MB4 and MB5 originate from GCB; subcluster MB3 exhibits features indicative of a dual origin from both ABC and GCB. The pseudotime trajectory clearly bifurcates into two major branches. The left branch contains MB1, MB2, and MB6 (all ABC‐origin), whereas the right branch contains MB4 and MB5 (GCB‐origin). MB3 sits exactly at the bifurcation point. This branching pattern indicates a differentiation process, with MB3 as a possible transitional state. These observations directly motivated our subsequent analyses comparing ABC‐ and GCB‐derived subgroups.

### 3.2. Cell–Cell Communication Analysis Among Different Cell Populations

**Figure 2:**
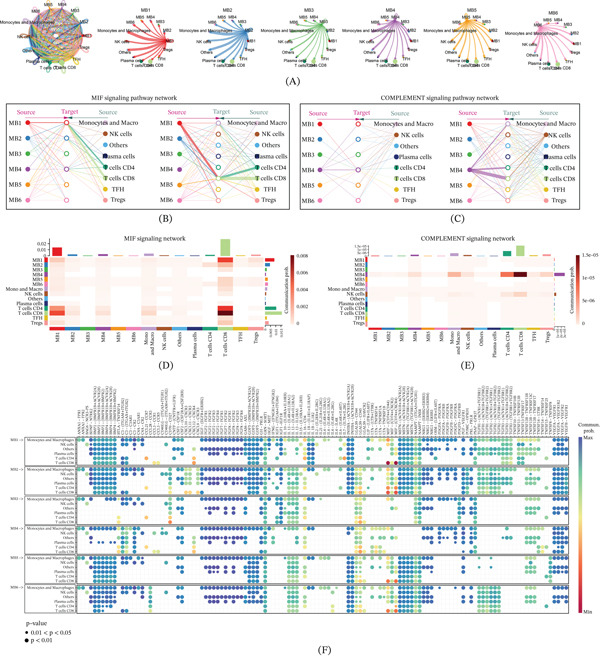
Cell–cell communication analysis within the DLBCL tumor microenvironment. (A) Bar plot showing the total communication signal strength between each of the six MB subpopulations and other major immune cell types. (B–C) Circle plots visualizing the outgoing and incoming signaling patterns of the MIF (B) and complement (C) pathways across different cell types. (D–E) Heatmaps representing the communication probability of the MIF (D) and complement (E) signaling pathways between sender and receiver cell types. (F) Bubble plot summarizing the significant ligand‐receptor interactions, categorized by sender (MB subpopulation) and receiver (other cell types).

We employed the CellChat algorithm to visualize the communication network among various cell subpopulations. Figure 2A illustrates the communication signal strength between six MB subpopulations and other immune cells, including monocytes/macrophages, NK, Treg, TFH, CD8+ T cells, plasma cells, and CD4+ T cells. Each subsequent panel in Figure 2A focuses on a specific MB subpopulation, detailing its communication interactions with other cell types. MB5 shows the strongest outgoing communication signals, especially to monocytes/macrophages and CD8+ T cells, indicating that MB5 may actively remodel the microenvironment. In contrast, MB1 and MB2 exhibit very weak interactions with immune cells, suggesting a relatively “immune silent” state. Figure 2B,C depict the signaling pathways of the MIF and complement across the six MB subpopulations and other cell types, respectively. These figures highlight the intensity of signaling interactions, providing insights into the predominant communication routes within the immune network. Line thickness represents predicted signaling strength (log‐normalized probability). For the MIF pathway (Figure 2B), thick lines from MB5 to macrophages are expected, as MIF is a known protumor cytokine in lymphoma. For the complement pathway (Figure 2C), we unexpectedly observed strong interactions between MB3 and plasma cells—a finding not previously reported in DLBCL, suggesting a novel crosstalk. To further quantify the communication probabilities between different cell types, Figure 2D,E present heatmaps for the MIF and complement signaling pathways, respectively, showing that MB5 is a strong “sender” for both MIF and complement signals (dark red squares). These heatmaps visually represent the communication probability, with color gradients indicating the likelihood of interactions between sender and receiver cells across various cell types. Figure 2F provides a comprehensive overview of ligand‐receptor interactions across different MB subpopulations and other immune cells, and identifies specific ligand‐receptor pairs, for example, MIF‐CD74 from MB5 to macrophages and C3‐CR1 from MB3 to plasma cells. This bubble plot categorizes interactions based on the senders (MB subpopulation) and receivers (other cell types), with bubble sizes and colors corresponding to the strength of the communication signals.

### 3.3. The MB5 Subgroup Signature Is the Most Critical Factor Affecting the Prognosis of DLBCL Patients

**Figure 3:**
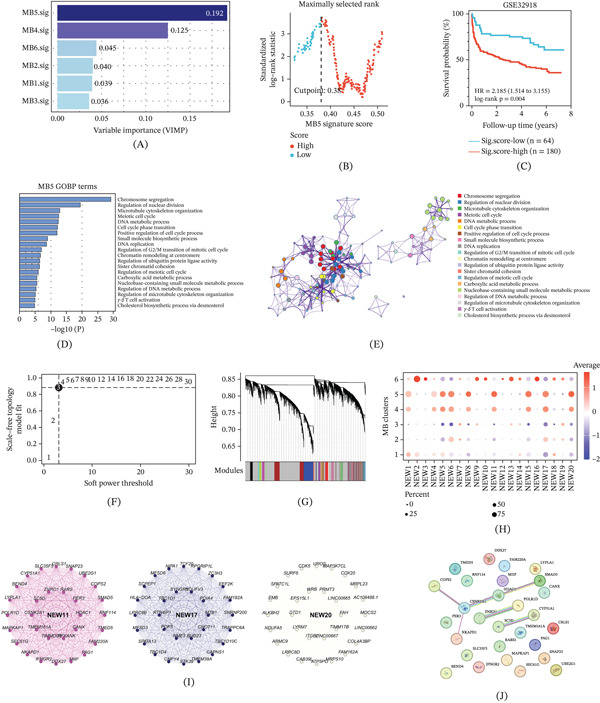
The MB5 subgroup signature as a critical prognostic factor in DLBCL. (A) Variable importance (VIMP) plot from a random survival forest model, ranking the prognostic value of the six MB subgroup signatures (GSVA scores). (B) Determination of the optimal cutoff (0.38) for the MB5 signature score using maximally selected rank statistics. (C) Kaplan–Meier survival curves comparing DLBCL patients stratified by high and low MB5 signature scores (log‐rank *p* = 0.0004). (D–E) Gene Ontology (GO) enrichment analysis of MB5 subgroup marker genes, displayed as a (D) bar plot and a (E) network plot. (F) Analysis of scale‐free topology fit index for selecting the soft‐thresholding power (power = 3) in hdWGCNA. (G) Hierarchical clustering dendrogram of genes and the identified coexpression modules. (H) Heatmap showing the correlation between module eigengenes and the six malignant B‐cell subclusters. (I) Visualization of coexpression networks for the three modules (NEW11, NEW17, and NEW20) most correlated with the MB5 subgroup. (J) Protein–protein interaction (PPI) network for core genes within the NEW11 module.

Subsequently, we aimed to evaluate the prognostic significance of different malignant B‐cell subgroups (MB1–MB6) in DLBCL by identifying signature genes for each subgroup using the FindAllMarkers function and selecting the Top 500 markers. We then applied GSVA to quantify these signatures in the GSE32918 cohort. Figure 3A shows that the MB5 subgroup had the highest predictive value for patient survival with a variable importance (VIMP) of 0.192. The maximally selected rank statistics analysis in Figure 3B identified a cutoff value of 0.38 for the MB5 signature score, which was used to stratify patients into high and low signature score groups. Figure 3C demonstrates that patients with high MB5 signature scores had significantly lower survival probabilities (HR = 2.185, 95% CI: 1.514–3.155, log‐rank *p* = 0.0004), indicating that the MB5 signature score is a crucial prognostic indicator for DLBCL patients. The MB5 subgroup marker genes were enriched in biological processes such as chromosome segregation and nuclear division, as shown in Figure 3D,E. In addition to chromosome segregation and nuclear division, a distinct cluster of genes involved in microtubule cytoskeleton organization is also enriched (green cluster in Figure 3E). This suggests that MB5 cells may have altered cytoskeletal dynamics, which could contribute to their high proliferative and invasive capacity—a hypothesis that now deserves experimental testing. To further explore the gene expression characteristics of the MB5 subgroup, we conducted WGCNA using the hdWGCNA algorithm. The optimal soft power threshold for the best model fit was determined to be 3 in Figure 3F. Figure 3G shows a dendrogram of the gene coexpression network, categorizing genes into multiple modules, with Figure 3H illustrating the correlation between these modules and each malignant B‐cell subgroup. The dendrogram shows hierarchical clustering of genes into coexpression modules (each assigned a color). The scale‐free topology fit index (Figure 3F) validated that a soft‐thresholding power of 3 produces a biologically meaningful network. Readers can see that the branches are cut at a height that yields discrete modules, which are then correlated with the MB subgroups in Figure 3H. NEW11, NEW17, and NEW20 showed the highest correlation with the MB5 subgroup, suggesting their potential key roles. Figure 3I provides coexpression gene network diagrams for these modules, highlighting core genes such as SLC35F6, SNAP23 in NEW11; NFATC1, TNFRSF1A in NEW17; and CDK5, URGCP in NEW20. These pathways are hallmarks of aggressive B‐cell lymphomas and align with MYC‐ and E2F‐driven programs defining the “proliferative” DLBCL subtype [15]. The NEW11 module includes SLC35F6 (cell‐cycle progression) and SNAP23 (cytokinesis), thus representing a core proliferation network. NEW17 contains NFATC1 and TNFRSF1A, linking MB5 to NF‐AT and TNF signaling—known survival pathways in ABC DLBCL. NEW20 contains CDK5 and URGCP, implicated in chemoresistance. Therefore, MB5 corresponds to a “proliferative and pro‐survival” signature. Figure 3J details the PPI network within the NEW11 module. Edges in the STRING network represent predicted physical or functional associations (combined score > 0.4). The relatively sparse connectivity among nodes in the NEW11 module (most nodes have only 1–2 edges) indicates that many hub genes may not directly interact at the protein level. Instead, they may function in parallel pathways or via indirect regulatory mechanisms—a common feature in proliferation‐associated transcriptional programs. Their biological relevance is supported by robust coexpression in the WGCNA analysis, which does not require direct physical interactions.

### 3.4. Distinct Mutation Patterns Were Observed in DLBCL Samples With High and Low MB5 Signature Scores

**Figure 4:**
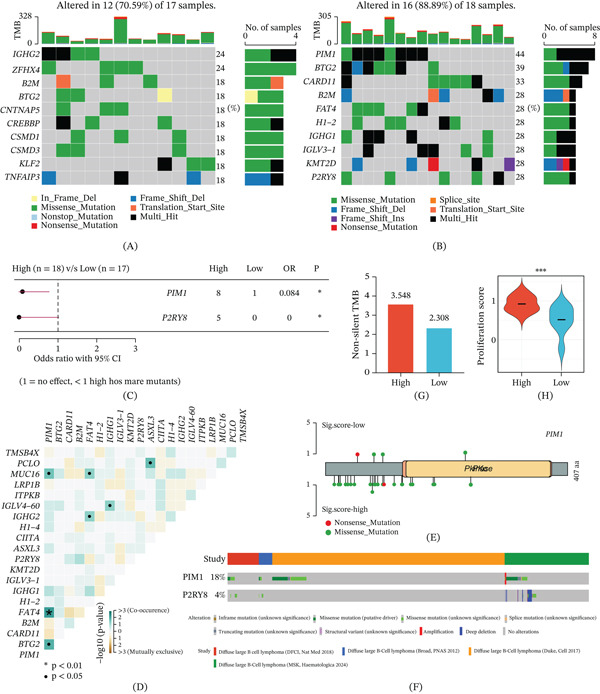
Distinct mutational patterns and increased tumor burden in DLBCL samples with high MB5 signature scores. (A–B) Oncoplots showing the mutation landscape of the Top 20 mutated genes in the (A) low and (B) high MB5 signature score groups from the TCGA‐DLBCL cohort. (C) Forest plot comparing the odds ratio (OR) of PIM1 and P2RY8 gene mutations between the two groups. (D) Interaction plot of significant co‐occurring mutations within the high MB5 score group. (E) Lollipop plot depicting the distribution and types of PIM1 mutations in both groups. (F) Summary of genomic alteration frequency and variant classification for PIM1 and P2RY8 across four DLBCL datasets. (G–H) Box plots comparing the (G) tumor mutational burden (TMB) and (H) proliferation score between the high and low MB5 score groups.

Oncoplots were generated to depict the mutations for samples with low and high MB5 signature scores in Figure 4A,B, respectively. Beyond simple mutation frequency, we observed meaningful co‐occurrence and mutual exclusivity. In the high‐MB5 group, PIM1 and BTG2 mutations frequently co‐occurred, whereas CARD11 and FAT4 were mutually exclusive (never together). In the low‐MB5 group, no such structured pattern was seen, indicating that high‐MB5 tumors follow specific oncogenic pathway dependencies. Figure 4C further compares the distribution of PIM1 and P2RY8 gene mutations between the two groups using a forest plot. The odds ratio (OR) for PIM1 gene mutation in the high‐scoring group is 0.084, and for P2RY8, it is 0, suggesting a significant enrichment of mutations in these genes within the high‐scoring MB5 subgroup. Figure 4D displays the comutation patterns within the high‐scoring group, highlighting that the PIM1/FAT4 comutation is a characteristic mutational feature of this group. PIM1 is an oncogenic kinase; FAT4 is a tumor suppressor in Hippo signaling. Their comutation (the most significant comutation pattern in the high‐risk group) suggests a “two‐hit” mechanism. Figure 4E presents a lollipop plot depicting the distribution of hotspot mutations in PIM1 across high and low‐scoring groups. The lollipop plot reveals that PIM1 mutations in the high‐MB5 group are concentrated in the kinase domain, especially at residue L221 (a known activating hotspot). In the low‐MB5 group, mutations are sporadic and located outside functional domains. This domain‐specific enrichment strongly suggests that PIM1 is a driver mutation in the MB5 subgroup, not a passenger. Figure 4F illustrates the genomic alteration frequency and variant distribution of the mutated genes PIM1 and P2RY8 across four different DLBCL datasets. PIM1 mutations are primarily missense type, whereas P2RY8 mutations are predominantly deep deletions. Figure 4G compares the nonsilent mutation burden (TMB) between the two groups, showing that the high‐scoring group has a significantly higher TMB (mean: 3.548 vs. 2.308), indicating a correlation between high MB5 subgroup scores and increased TMB. Additionally, the proliferation score is significantly higher in the high‐scoring group (*p* < 0.0001; Figure 4H), suggesting a link between high MB5 subgroup scores and enhanced tumor cell proliferation.

### 3.5. Development and Validation of a Prognostic Machine Learning Model for Predicting Overall Survival in DLBCL Patients

**Figure 5:**
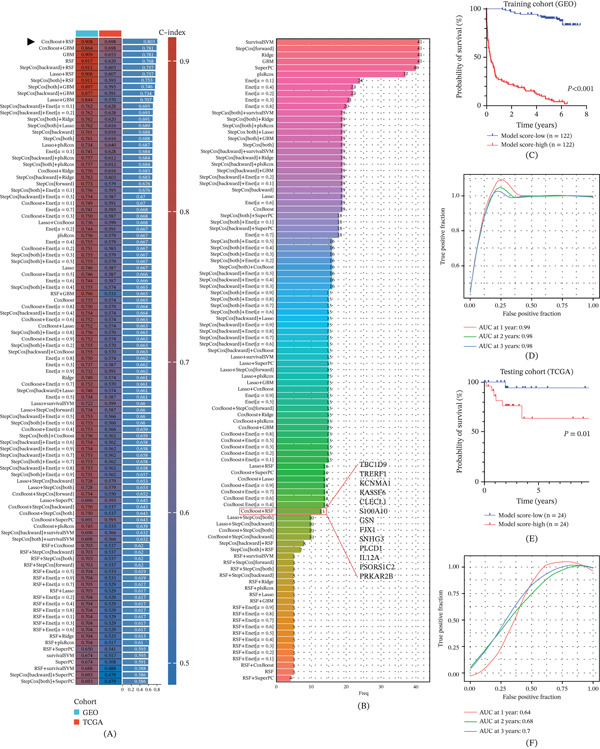
Development and validation of a machine learning prognostic model for DLBCL. (A) Bar plot of the concordance index (C‐index) for 101 machine learning ensembles tested on the training (GSE32918) and validation (TCGA‐DLBCL) cohorts, highlighting the top‐performing CoxBoost‐RSF model. (B) The number of genes used in each of the 101 machine learning models. (C) Kaplan–Meier survival curves for patients stratified into high‐ and low‐risk groups by the CoxBoost‐RSF model in the GSE32918 training cohort. (D) Time‐dependent receiver operating characteristic (ROC) curves for 1‐, 2‐, and 3‐year overall survival prediction in the GSE32918 cohort. (E–F) Kaplan–Meier curves (E) and time‐dependent ROC curves (F) for the CoxBoost‐RSF model in the TCGA‐DLBCL validation cohort.

Next, we constructed prognostic models using 101 machine learning ensembles based on MB5 marker genes, with GSE32918 serving as the training cohort and TCGA‐DLBCL as the testing cohort. Among these, the CoxBoost‐RSF ensemble demonstrated the highest C‐index (GSE32918 C − index = 0.908; TCGA C − index = 0.698; Figure 5A). Figure 5B illustrates the number of genes included in each machine learning model, with the optimal CoxBoost‐RSF model comprising 13 genes. Figure 5C presents the Kaplan–Meier curves for the high‐ and low‐risk groups based on the CoxBoost‐RSF model risk scores in the GSE32918 dataset, whereas Figure 5D shows the 1‐, 2‐, and 3‐year ROC curves for the same dataset. Figure 5E depicts the KM curves for the high‐ and low‐risk groups in the TCGA‐DLBCL dataset, and Figure 5F presents the 1‐, 2‐, and 3‐year ROC curves for this dataset. The Kaplan–Meier curves show a clear and sustained separation between high‐ and low‐risk groups. Median overall survival of the high‐risk group is much shorter than that in the low‐risk group. Time‐dependent AUC values in the training cohort are 0.98 at 2 and 3 years, and 0.68 at 2 years and 0.7 at 3 years in the testing cohort, which indicates a good discriminative power over time. Clinically, this separation could guide treatment intensification—for example, high‐risk patients might require more intensive surveillance and aggressive treatment.

### 3.6. Distinct Immune Landscapes and Differential Drug Sensitivity Between Different Risk Groups

**Figure 6:**
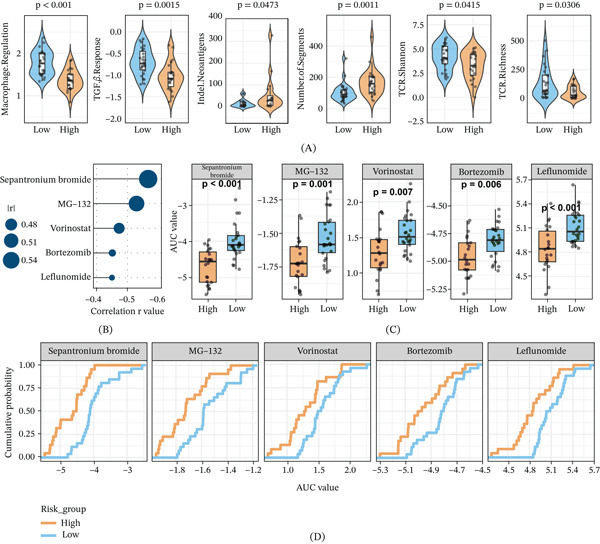
Immune microenvironment characterization and drug sensitivity profiling of the prognostic risk groups. (A) Violin plots show six significantly differential immune features between the low‐risk and high‐risk groups, including macrophage regulation, TGF‐*β* response, indel neoantigens, number of segments, TCR Shannon diversity, and TCR richness. (B) Correlation analysis between risk scores and estimated IC50 values (AUC) of candidate therapeutic compounds. The top five agents with the strongest negative correlations (Sepantronium bromide, MG‐132, vorinostat, bortezomib, and leflunomide) are presented, with bubble size proportional to the absolute correlation coefficients. (C) Box plots compare estimated IC50 values (AUC) of the five indicated drugs between low‐risk and high‐risk groups. (D) Cumulative probability plots of estimated IC50 values for the five compounds between the two risk groups.

Based on the prognostic stratification of the TCGA‐DLBCL cohort, we next sought to characterize the immune microenvironment and therapeutic sensitivity profiles associated with the low‐risk and high‐risk subgroups (Figure 6). As depicted in Figure 6A, six immune‐related features were significantly differentially represented between the two groups. The low‐risk group exhibited significantly higher scores for macrophage regulation (*p* < 0.001), TGF‐*β* response (*p* = 0.0015), TCR Shannon diversity (*p* = 0.0415), and TCR richness (*p* = 0.0306). In contrast, the high‐risk group displayed significantly elevated indel neoantigen burden (*p* = 0.0473) and an increased number of segments (*p* = 0.0011), underscoring distinct immunogenomic attributes linked to prognostic risk.

To explore whether the risk score could inform therapeutic response, we performed correlation analyses between the risk score and estimated IC50 values (AUC) of various compounds. As shown in Figure 6B, significant negative correlations were identified for multiple agents, with the five most negative associations observed for Sepantronium bromide (*r* = –0.5670), MG‐132 (*r* = –0.5296), vorinostat (*r* = –0.4733), bortezomib (*r* = –0.4515), and leflunomide (*r* = –0.4508). These findings indicate that higher risk scores corresponded to greater drug sensitivity.

This trend was further validated by direct group comparisons of IC50 estimates (Figure 6C). The high‐risk group consistently exhibited significantly lower AUC values than the low‐risk group across all five agents: Sepantronium bromide (*p* < 0.001), MG‐132 (*p* = 0.001), vorinostat (*p* = 0.007), bortezomib (*p* = 0.006), and leflunomide (*p* < 0.001). Finally, cumulative probability plots confirmed these observations, revealing a pronounced leftward shift in the distribution curves of the high‐risk group for each drug, thereby robustly establishing enhanced chemosensitivity in the high‐risk prognostic subgroup (Figure 6D).

## 4. Discussion

DLBCL is a highly heterogeneous disease, both clinically and molecularly [31, 32]. This heterogeneity is reflected in the diverse symptoms, disease progression rates, and treatment responses observed among DLBCL patients [31]. Our study leverages scRNA‐seq data to explore this heterogeneity, focusing on identifying distinct subgroups of malignant B cells and evaluating their prognostic significance. The findings provide valuable insights into the molecular underpinnings of DLBCL and highlight potential therapeutic targets.

The identification of six distinct malignant B‐cell subclusters (MB1–MB6) through scRNA‐seq analysis underscores the cellular heterogeneity within DLBCL tumors. The functional differences between subclusters are directly demonstrated by GO enrichment. For example, MB3 uniquely expressed complement‐related genes, MB5 upregulated proliferation pathways, and MB6 showed B‐cell receptor signaling enrichment. The pseudotime analysis further reveals the dynamic relationships among these subclusters, providing a temporal perspective on cellular differentiation and development. This information is crucial for understanding the progression of DLBCL and identifying potential therapeutic intervention points.

The CellChat algorithm‐based cell–cell communication analysis provides a comprehensive view of the interactions within the tumor microenvironment. The communication network among different cell populations, including malignant B cells and various immune cells, highlights the complexity of the signaling pathways involved. The identification of specific signaling pathways, such as MIF and complement [33, 34], along with their communication probabilities between different cell types, provides insights into the complex immune network within the DLBCL tumor microenvironment. The cell–cell interaction analysis quantitatively showed that the MIF pathway is the most active route from MB5 to macrophages, and the complement pathway from MB3 to plasma cells (Figure 2B–F). MB5 cells are the strongest senders of MIF signals, primarily targeting macrophages. MIF‐CD74 signaling can polarize macrophages toward an M2‐like immunosuppressive phenotype, thereby creating a tumor‐permissive microenvironment. Similarly, the complement pathway, particularly C3‐CR1 interactions between MB3 and plasma cells, may enhance malignant B‐cell survival by promoting resistance to complement‐dependent cytotoxicity. These findings suggest that targeting MIF or complement components could disrupt protumor crosstalk in DLBCL. These findings are consistent with previous reports that MIF promotes immunosuppression and complement enhances B‐cell survival [34].

The MB5 subgroup emerges as a critical factor affecting the prognosis of DLBCL patients. The high predictive value of the MB5 signature score for patient survival, as demonstrated by GSVA, indicates its potential as a prognostic marker. The enrichment of MB5 marker genes in biological processes such as chromosome segregation and nuclear division suggests a role in tumor cell proliferation. Beyond GO terms such as chromosome segregation, we speculate that the aggressiveness of the MB5 subgroup may be driven by several upstream transcriptional regulators. The coexpression module NEW11 includes multiple MYC target genes, whereas the enrichment of proliferation markers suggests E2F family activation. Furthermore, the presence of PIM1 mutations and TNFRSF1A expression points to aberrant NF‐*κ*B signaling, a known survival pathway in DLBCL. Thus, MB5 likely represents a state codriven by MYC/E2F proliferative programs and NF‐*κ*B‐mediated survival signals. The WGCNA further identifies core modules and hub genes associated with the MB5 subgroup, providing a detailed understanding of its gene expression characteristics. These findings highlight the MB5 subgroup as a potential therapeutic target, as targeting its specific genetic and cellular features could improve patient outcomes.

The analysis of TMB and proliferation capacity in the TCGA‐DLBCL cohort reveals a significant correlation between high MB5 subgroup scores and increased TMB and proliferation scores. This suggests that the MB5 subgroup is associated with enhanced tumor cell proliferation capacity and genetic instability. The distinct mutation patterns observed in samples with high and low MB5 signature scores, including the enrichment of mutations in genes such as PIM1 and P2RY8 in the high‐scoring group, further emphasize the molecular differences between these groups. These mutations may contribute to the aggressive nature of DLBCL and could be potential targets for therapeutic intervention in high‐risk DLBCL patients [17, 35].

The development and validation of prognostic machine learning models based on MB5 marker genes demonstrate the potential for improving survival prediction in DLBCL patients. The CoxBoost‐RSF ensemble model, with its high C‐index values in both the training and testing cohorts, shows promise as a robust prognostic tool. The inclusion of specific marker genes in the model highlights their importance in predicting patient outcomes. The Kaplan–Meier curves and ROC curves further validate the model′s effectiveness in stratifying patients into high‐ and low‐risk groups.

Several limitations should be acknowledged. First, our single‐cell analysis relied on a single public dataset (GSE182434); validation in additional independent scRNA‐seq cohorts is needed to confirm the MB1‐MB6 subclusters. Second, the prognostic model′s C‐index in the TCGA validation set is modest, which may be improved by incorporating clinical variables (e.g., IPI score) or additional genomic features. Third, the functional roles of the PIM1/FAT4 comutation have not been experimentally tested. Future work should include CRISPR‐based perturbations in DLBCL cell lines and xenograft models to establish causality. Fourth, our model predicts overall survival but not response to specific therapies (e.g., R‐CHOP vs. pola‐R‐CHP); this represents a key direction for clinical translation. Finally, prospective cohort studies are required before the MB5 signature can be used in routine diagnostics.

In conclusion, our study provides a comprehensive analysis of the cellular and molecular heterogeneity within DLBCL, identifying the MB5 subgroup as a critical factor affecting patient prognosis. The findings highlight the potential of the MB5 subgroup as a therapeutic target and emphasize the importance of personalized treatment strategies based on the specific genetic and cellular features of each patient′s tumor. Future studies should validate these findings in larger cohorts and explore the therapeutic potential of targeting the MB5 subgroup and its associated signaling pathways. Additionally, further investigation into the functional roles of the identified hub genes and their interactions within the tumor microenvironment could provide valuable insights into the pathogenesis of DLBCL and lead to the development of novel therapeutic approaches.

## 5. Conclusion

Our study utilized scRNA‐seq to delineate cellular heterogeneity in DLBCL, identifying six malignant B‐cell subclusters. The MB5‐related gene signature was recognized as a critical prognostic factor associated with poor survival, higher TMB, enhanced proliferation, and specific mutations. Analysis of cell–cell communication uncovered relevant signaling pathways within the tumor microenvironment. Furthermore, prognostic machine learning models were successfully developed and validated in DLBCL patients using MB5 signature genes. These findings highlight MB5 as a promising therapeutic target and provide a framework for advancing personalized risk stratification and treatment strategies in DLBCL.

## Author Contributions

Hongyu Shen and Lei Fan conceived and designed the study. Jinbo Lu and Qi Yan contributed to data collection and analysis. Jinjiang Chou and Xiao Liang wrote the original draft. Weifei Fan provided critical revisions and contributed to the paper′s editing. Hongyu Shen, Jinbo Lu, and Qi Yan contributed equally to this work.

## Funding

This study was supported by The Natural Science Foundation of Jiangsu Province (BK20232039); Jiangsu Province Hospital (the First Affiliated Hospital with Nanjing Medical University) Clinical Capacity Enhancement Project (JSPH‐MB‐2021‐9); Clinical Diagnosis and Treatment Technology Innovation Open Bidding and Leader Selecting Project of Jiangsu Province Hospital (JBGS202404); National Natural Science Foundation of China (Grant No. 82470186); The Specialized Diseases Clinical Research Fund of Jiangsu Province Hospital (XB 202401); and the Jiangsu Funding Program for Excellent Postdoctoral Talent (2025ZB699).

## Disclosure

All authors read the final version and approved the submission.

## Ethics Statement

This study is based on published or publicly available datasets and does not require ethical approval or consent.

## Conflicts of Interest

The author declares no conflicts of interest.

## Data Availability

All presented data and relevant codes in this study are available from the corresponding author upon reasonable request.
